# The Double-High Phenotype: Synergistic Impact of Metabolic and Arterial Load on Ambulatory Blood Pressure Instability

**DOI:** 10.3390/jcm15020872

**Published:** 2026-01-21

**Authors:** Ahmet Yilmaz, Azmi Eyiol

**Affiliations:** 1 Department of Cardiology, Faculty of Medicine, Karamanoğlu Mehmetbey University, Karaman 70200, Turkey; 2 Department of Cardiology, Beyhekim Training and Research Hospital, Konya 42100, Turkey; azmieyiol@yahoo.com

**Keywords:** ambulatory blood pressure monitoring (ABPM), arterial stiffness, blood pressure variability, hypertension, triglyceride–glucose index (TyG)

## Abstract

**Background/Objectives:** Insulin resistance and ambulatory blood pressure monitoring (ABPM) abnormalities represent distinct but interrelated pathways contributing to cardiovascular risk. The triglyceride–glucose (TyG) index reflects metabolic burden, whereas arterial load—captured through arterial stiffness, blood pressure variability, and morning surge—reflects hemodynamic instability. Whether the coexistence of these domains identifies a particularly high-risk ambulatory phenotype remains unclear. To evaluate the independent and combined effects of metabolic burden (TyG) and arterial load on circadian blood pressure pattern and short-term systolic blood pressure variability. **Methods:** This retrospective cross-sectional study included 294 adults who underwent 24 h ABPM. Arterial load was defined using three ABPM-derived indices (high AASI, high SBP-ARV, high morning surge). High metabolic burden was defined as TyG in the upper quartile. The “double-high” phenotype was classified as high TyG plus high arterial load. Primary and secondary outcomes were non-dipping pattern and high SBP variability. Multivariable logistic regression and Firth penalized models were used to assess independent associations. Predictive performance was evaluated using ROC analysis. **Results:** The double-high phenotype (*n* = 15) demonstrated significantly higher nighttime SBP, reduced nocturnal dipping, and markedly elevated BP variability. It was the strongest independent predictor of non-dipping (adjusted OR = 42.0; Firth OR = 11.73; both *p* < 0.001) and high SBP variability (adjusted OR = 41.7; Firth OR = 26.29; both *p* < 0.001). Arterial load substantially improved model discrimination (AUC = 0.819 for non-dipping; 0.979 for SBP variability), whereas adding TyG to arterial load produced minimal incremental benefit. **Conclusions:** The coexistence of elevated TyG and increased arterial load defines a distinct hemodynamic endotype characterized by severe circadian blood pressure disruption and exaggerated short-term variability. While arterial load emerged as the principal determinant of adverse ambulatory blood pressure phenotypes, TyG alone demonstrated limited discriminative capacity. These findings suggest that TyG primarily acts as a metabolic modifier, amplifying adverse ambulatory blood pressure phenotypes predominantly in the presence of underlying arterial instability rather than serving as an independent discriminator. Integrating metabolic and hemodynamic domains may therefore improve risk stratification and help identify a small but clinically meaningful subgroup of patients with extreme ambulatory blood pressure dysregulation.

## 1. Introduction

Hypertension is one of the leading causes of global cardiovascular morbidity and mortality, affecting more than one billion individuals worldwide [1]. Although office blood pressure measurements provide important diagnostic information, ambulatory blood pressure monitoring (ABPM) offers superior risk stratification by capturing circadian blood pressure patterns and short-term variability [2]. Among these ambulatory phenotypes, non-dipping status, exaggerated morning surge, and increased blood pressure variability are strongly associated with target-organ damage, arterial stiffness, and adverse cardiovascular outcomes [3,4,5]. These high-risk ambulatory patterns reflect an advanced stage of vascular dysfunction, arterial stiffening, and impaired autonomic regulation rather than isolated hemodynamic abnormalities [6].

Insulin resistance plays a central role in the development and progression of hypertension through multiple interrelated mechanisms, including sympathetic nervous system activation, endothelial dysfunction, oxidative stress, and maladaptive vascular remodeling [7,8]. Although the hyperinsulinemic-euglycemic clamp remains the gold standard for assessing insulin resistance, its complexity and limited feasibility restrict its routine clinical application. Consequently, the triglyceride–glucose (TyG) index has emerged as a simple, reproducible, and cost-effective surrogate marker of insulin resistance. Accumulating evidence from large-scale cohort studies and meta-analyses has consistently demonstrated that elevated TyG levels are independently associated with an increased risk of incident hypertension, adverse cardiometabolic outcomes, and subclinical vascular dysfunction, beyond traditional cardiovascular risk factors [9,10,11].

Beyond blood pressure elevation alone, TyG also appears to be closely linked to adverse vascular structure and function. In hypertensive populations, higher TyG levels have been shown to correlate independently with increased arterial stiffness, as assessed by brachial–ankle pulse wave velocity, even after comprehensive adjustment for metabolic confounders. Population-based studies further demonstrate that elevated TyG is associated with both uncontrolled hypertension and higher estimated pulse wave velocity, suggesting that metabolic insulin resistance contributes directly to progressive arterial stiffening and impaired vascular compliance. Collectively, these findings support the concept that TyG reflects not only metabolic dysregulation but also the cumulative structural burden imposed on the vascular wall [12,13,14,15].

Importantly, emerging evidence indicates that TyG is linked not only to static blood pressure levels but also to adverse circadian blood pressure phenotypes assessed by ABPM. In newly diagnosed, treatment-naïve hypertensive patients, higher TyG values have been independently associated with a non-dipping blood pressure pattern, with TyG outperforming fasting glucose, triglycerides, and even HOMA-IR in discriminating non-dippers [16,17]. These observations provide mechanistic support for a close interaction between metabolic insulin resistance and abnormal nocturnal autonomic–vascular regulation. In contrast, ABPM-derived abnormalities—such as non-dipping status, exaggerated morning surge, reduced smoothness index, and increased short-term blood pressure variability—primarily capture the hemodynamic instability and arterial load imposed on the vasculature over a 24 h period.

Despite the growing body of literature linking TyG to hypertension, arterial stiffness, and non-dipping patterns individually, the combined impact of metabolic burden and arterial load on high-risk ambulatory blood pressure phenotypes has not been systematically investigated. In clinical reality, cardiometabolic risk rarely arises from a single pathway; rather, it reflects the convergence of metabolic insulin resistance and cumulative arterial stress. Whether the coexistence of high metabolic burden, as reflected by elevated TyG, and high arterial burden, as reflected by adverse ABPM-derived indices, identifies a subgroup of patients with particularly unfavorable ambulatory blood pressure behavior remains largely unknown.

Conceptually, metabolic burden assessed by the TyG index may reflect a background vulnerability that predisposes individuals to vascular dysfunction, whereas ABPM-derived arterial load captures the functional hemodynamic expression of this risk. The proposed “double-high” phenotype therefore represents a synergistic interaction between metabolic susceptibility and arterial instability rather than a merely additive combination of risk factors. In real-world practice, cardiometabolic risk frequently arises from the convergence of these pathways rather than from a single mechanism. Understanding the interaction between metabolic load (TyG) and arterial load (ABPM-derived indices) may therefore provide a more comprehensive framework for identifying patients at extreme risk for circadian and short-term blood pressure dysregulation.

Accordingly, the present study aimed to evaluate whether the combination of elevated TyG and increased arterial load—the “double-high phenotype”—is associated with a markedly higher prevalence of non-dipping status and increased systolic blood pressure variability compared with either burden alone. By integrating metabolic and hemodynamic domains, we sought to explore the synergistic clustering of cardiometabolic risk in patients undergoing ABPM.

## 2. Methods

### 2.1. Study Design and Population

This single-center, retrospective, cross-sectional observational study included adult patients who underwent 24 h ambulatory blood pressure monitoring (ABPM) between January 2022 and September 2025 at the Cardiology Department of Karaman Training and Research Hospital. Clinical, laboratory, and ABPM data were obtained from the institutional electronic medical records. The study was conducted in accordance with the Declaration of Helsinki and approved by the Clinical Research Ethics Committee of Karamanoğlu Mehmetbey University Faculty of Medicine (date: 16 October 2025, decision no: 24-2025/21). Because of the retrospective design, informed consent was waived. All data were anonymized.

### 2.2. Inclusion and Exclusion Criteria

Eligible participants were adults aged ≥ 18 years with valid 24 h ABPM recordings (≥70% valid measurements) and concurrent fasting triglyceride and glucose analyses. Exclusion criteria included secondary hypertension, acute coronary syndrome, heart failure, severe systemic disease, acute infection, thyroid dysfunction, electrolyte imbalance, pregnancy, invalid ABPM data, or missing laboratory results.

### 2.3. Ambulatory Blood Pressure Monitoring

ABPM was performed using a validated oscillometric device (Mobil-O-Graph, IEM GmbH, Stolberg, Germany), programmed for automated measurements every 15 min during daytime and every 30 min during nighttime. ABPM was performed under usual clinical conditions. For patients receiving antihypertensive therapy, recordings were obtained while on stable medication regimens without recent dose changes; however, the exact timing of medication intake relative to ABPM initiation was not protocolized.

According to the 2024 ESC Hypertension Guidelines [17], ambulatory hypertension was defined as a mean 24 h systolic blood pressure (SBP) ≥ 130 mmHg and/or diastolic blood pressure (DBP) ≥ 80 mmHg, daytime hypertension as a mean SBP ≥ 135 mmHg and/or DBP ≥ 85 mmHg, and nighttime hypertension as a mean SBP ≥ 120 mmHg and/or DBP ≥ 70 mmHg. Based on ABPM recordings, circadian blood pressure pattern and variability metrics were evaluated as follows: a non-dipping pattern was defined as a nocturnal SBP reduction of less than 10%; blood pressure variability was assessed using the 24 h systolic BP standard deviation (SD) and average real variability (ARV); morning surge was calculated as the difference between the mean SBP during the first two hours after awakening and the lowest nighttime SBP; the smoothness index (SI) was derived from the 24 h SBP profile; and the arterial stiffness surrogate index (AASI) was calculated from the regression slope of DBP on SBP based on ABPM recordings.

Fasting plasma glucose and triglyceride levels were obtained from routine biochemical analyses. The triglyceride–glucose (TyG) index was calculated using the following formula:

*TyG* = ln[(*Triglyceride* (mg/dL) × *Glucose* (mg/dL))/2]

The TyG index was analyzed as a continuous variable, as a z-score–standardized variable (TyG_z) for combined burden analyses, and as quartiles (Q1–Q4) for categorical comparisons.

Arterial burden was defined using ABPM-derived vascular stiffness and hemodynamic instability parameters, including high ambulatory arterial stiffness index (AASI), low smoothness index, and excessive morning blood pressure surge. To avoid circularity and incorporation bias, non-dipping status and high systolic blood pressure variability were not included in the arterial load score and were exclusively analyzed as outcome variables. Each component contributed one point, yielding an arterial load score ranging from 0 to 3. Patients were classified as having low arterial load (0–1 points) or high arterial load (2–3 points). To quantify the combined cardiometabolic burden, a continuous composite score was calculated by summing the standardized TyG index (TyG_z) and the arterial load. For interaction-based categorical analyses, participants were further stratified into four groups according to metabolic and arterial burden status: low TyG–low arterial load, high TyG–low arterial load, low TyG–high arterial load, and high TyG–high arterial load (double-high phenotype).

These indices were selected because they represent complementary and clinically validated dimensions of arterial and autonomic dysfunction captured by ABPM: arterial stiffness (AASI), short-term hemodynamic instability (systolic BP variability), and circadian sympathetic activation (morning surge). Together, they provide a pragmatic and reproducible functional assessment of arterial load using routinely available ABPM parameters. This composite was intentionally constructed as an unweighted phenotype-based score to reflect the cumulative presence of adverse ABPM functional features rather than to impose assumptions about their relative contributions.

### 2.4. Study Endpoints

The primary outcome was non-dipping blood pressure pattern. Secondary endpoints included high systolic blood pressure variability (high ARV) and exaggerated morning surge.

### 2.5. Statistical Analysis

All analyses were performed using IBM SPSS Statistics v26 and R v4.3.0. Continuous variables were expressed as the mean ± standard deviation or median (interquartile range), and categorical variables as number and percentage. Group comparisons were performed using ANOVA or Kruskal–Wallis tests for continuous variables and chi-square test for categorical variables.

Multivariable logistic regression analyses were constructed using a hierarchical modeling strategy. Model 1 included age, sex, diabetes, and antihypertensive treatment as baseline clinical covariates. In Model 2, standardized TyG index (TyG_z) was added to evaluate its independent metabolic contribution. In Model 3, the arterial load score (high AASI + high SBP-ARV + high morning surge) was further incorporated to determine the incremental predictive value of arterial burden beyond clinical and metabolic factors. Non-dipping pattern and high systolic blood pressure variability were analyzed as separate dependent variables. Odds ratios (ORs) with 95% confidence intervals (CIs) were reported.

To evaluate the joint effect of metabolic and arterial burden, an interaction term between TyG_z and arterial load score was included in the multivariable model. In addition, categorical interaction was assessed using the four-group double-high phenotype classification, with the low–low group as the reference.

Receiver operating characteristic (ROC) curve analysis was performed to compare the discriminative performance of:

Model A: Clinical variables only;

Model B: Clinical variables + TyG_z;

Model C: Clinical variables + arterial load score (high AASI + high SBP-ARV + high morning surge);

Model D: Clinical variables + TyG_z + arterial load score (high AASI + high SBP-ARV + high morning surge).

The area under the curve (AUC) values were compared using the DeLong test to determine whether the combined model provided superior classification of non-dipping and high BP variability.

Given the presence of quasi-complete separation in some models, penalized maximum likelihood estimation (Firth regression) was additionally performed to ensure unbiased parameter estimation.

Statistical significance was defined as *p* < 0.05.

## 3. Results

### 3.1. Baseline Characteristics

A total of 294 patients were included in the final analysis. The mean age of the study population was 51.8 ± 11.7 years, and 49.7% were male. Diabetes mellitus was present in 8.8% of participants, while 38.8% were receiving antihypertensive treatment at the time of ABPM. The mean fasting plasma glucose level was 98.3 ± 15.5 mg/dL, mean triglyceride level was 155.8 ± 59.9 mg/dL, and the mean TyG index was 8.86 ± 0.41. Baseline demographic, metabolic, and clinical characteristics are summarized in Table 1.

### 3.2. Ambulatory Blood Pressure and Variability Parameters

Mean 24 h systolic and diastolic blood pressures were 131.4 ± 11.7 mmHg and 79.2 ± 8.3 mmHg, respectively. The average nocturnal systolic blood pressure dipping was 12.0 ± 5.4%. The mean ambulatory arterial stiffness index (AASI) was 0.45 ± 0.09, systolic blood pressure average real variability (SBP-ARV) was 12.23 ± 3.16 mmHg, and diastolic blood pressure ARV was 8.76 ± 2.38 mmHg. The smoothness index averaged 1.10 ± 0.11, and the mean morning blood pressure surge was 17.5 ± 5.6 mmHg (Table 1).

The prevalence of abnormal ambulatory phenotypes was as follows: non-dipping pattern in 29.6%, high SBP variability (ARV) in 29.6%, elevated AASI in 15.3%, and exaggerated morning surge in 12.2% of patients. TyG quartiles were evenly distributed across the cohort (Q1–Q4: 25% each) (Table 1).

### 3.3. Comparison Between Double-High and Non–Double-High Phenotypes

The double-high phenotype, defined by the coexistence of elevated TyG and high arterial load, was identified in 15 patients. Compared with the non-double-high group, patients with the double-high phenotype exhibited significantly worse metabolic profiles, including higher fasting glucose levels (117.4 ± 24.3 vs. 97.2 ± 13.8 mg/dL), higher triglyceride levels (227.4 ± 64.6 vs. 151.0 ± 57.6 mg/dL), and a markedly higher TyG index (9.42 ± 0.28 vs. 8.82 ± 0.40; all *p* < 0.001) (Table 2).

Ambulatory hemodynamic parameters were also more severely impaired in the double-high group. Although 24 h systolic blood pressure tended to be higher (137.1 ± 10.9 vs. 131.0 ± 11.7 mmHg, *p* = 0.051), nighttime systolic blood pressure was significantly elevated (126.0 ± 13.1 vs. 114.2 ± 10.9 mmHg, *p* < 0.001), accompanied by a significantly blunted nocturnal dipping percentage (8.0 ± 5.0% vs. 12.7 ± 5.0%, *p* = 0.001) (Table 2).

Indices of arterial stiffness and blood pressure variability were markedly higher in the double-high phenotype. AASI (0.55 ± 0.06 vs. 0.44 ± 0.08, *p* < 0.001), SBP-ARV (15.69 ± 1.89 vs. 11.80 ± 2.98 mmHg, *p* < 0.001), DBP-ARV (10.16 ± 2.26 vs. 8.57 ± 2.33 mmHg, *p* = 0.011), and morning surge (23.62 ± 5.41 vs. 16.95 ± 5.29 mmHg, *p* < 0.001) were all significantly increased (Table 2).

Correspondingly, the prevalence of abnormal ABPM phenotypes was substantially higher in the double-high group, including non-dipping status (73.3% vs. 27.2%), high SBP variability (93.3% vs. 26.2%), and exaggerated morning surge (60.0% vs. 9.7%) (all *p* < 0.001) (Table 2).

### 3.4. Predictors of Non-Dipping Pattern

In multivariable logistic regression analysis adjusted for age, sex, diabetes mellitus, and antihypertensive treatment, the double-high phenotype emerged as the strongest independent predictor of a non-dipping blood pressure pattern (OR = 42.0, 95% CI 5.18–341.0, *p* < 0.001). None of the conventional clinical covariates reached statistical significance (Table 3).

Penalized Firth logistic regression confirmed the robustness of this association (OR = 11.73, 95% CI 3.61–47.63, *p* < 0.001) (Table 4).

### 3.5. Predictors of High Systolic Blood Pressure Variability

Similarly, in multivariable logistic regression analysis for high SBP variability, the double-high phenotype was the strongest independent predictor (OR = 41.7, 95% CI 6.02–289.3, *p* < 0.001), whereas no traditional clinical variable independently predicted this outcome (Table 5).

Firth regression yielded consistent results (OR = 26.29, 95% CI 6.15–246.70, *p* < 0.001) (Table 6).

### 3.6. Discriminative Performance of Predictive Models

**Figure 1:**
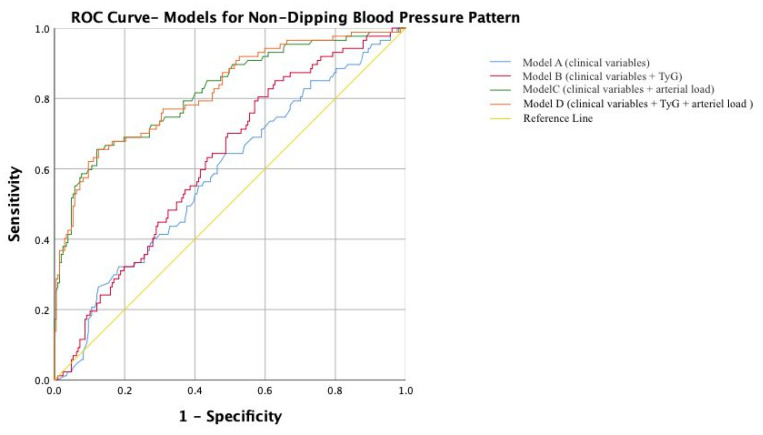
Receiver operating characteristic (ROC) curves comparing Model A (clinical variables), Model B (clinical variables + TyG), Model C (clinical variables + arterial load), and Model D (clinical variables + TyG + arterial load) for predicting the non-dipping blood pressure pattern.

For the prediction of non-dipping status, the clinical model demonstrated limited discrimination (AUC = 0.585). Models incorporating TyG alone modestly improved discrimination (AUC = 0.623), whereas arterial load parameters provided substantial discriminatory power (AUC = 0.819). The full model integrating clinical variables, TyG, and arterial load achieved an AUC of 0.821 (Table 7, Figure 1).

**Figure 2:**
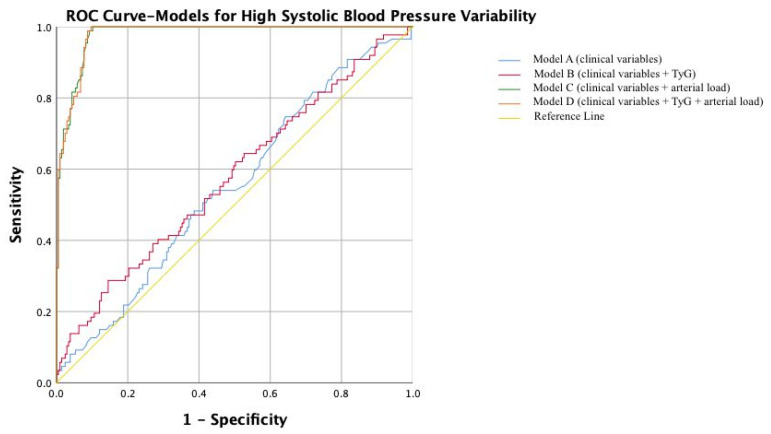
Receiver operating characteristic (ROC) curves comparing Model A (clinical variables), Model B (clinical variables + TyG), Model C (clinical variables + arterial load), and Model D (clinical variables + TyG + arterial load) for predicting high 24 h systolic blood pressure variability.

Similarly, for high SBP variability, the clinical model (AUC = 0.551) and TyG model (AUC = 0.577) showed poor discrimination, while the arterial load model demonstrated excellent performance (AUC = 0.979). The full model yielded a comparable AUC of 0.978 (Table 7, Figure 2).

DeLong test comparisons confirmed that arterial load parameters significantly improved model discrimination, whereas the addition of TyG to the arterial load model did not confer a meaningful incremental gain.

## 4. Discussion

In this ABPM-based study, we demonstrated that the coexistence of elevated metabolic burden (high TyG index) and increased arterial load identifies a distinct hemodynamic phenotype characterized by markedly impaired circadian blood pressure regulation and exaggerated short-term variability. Although TyG has been widely recognized as a surrogate marker of insulin resistance and a predictor of cardiometabolic disorders, our findings indicate that its impact on ambulatory blood pressure patterns becomes clinically meaningful predominantly when accompanied by functional arterial instability.

The association between TyG and blood pressure dysregulation has been previously reported, with several studies showing that higher TyG values are related to incident hypertension, endothelial dysfunction, and arterial stiffening [18,19,20,21]. However, the magnitude of these associations has generally been modest, and the ability of TyG to discriminate high-risk ABPM phenotypes has remained limited. Consistent with earlier observations, TyG alone showed only weak discriminatory power in our cohort, suggesting that metabolic stress may be associated with ambulatory BP instability primarily in conjunction with other hemodynamic or vascular factors rather than acting as a direct trigger on its own [22].

In contrast, arterial load emerged as the dominant determinant of both non-dipping status and systolic BP variability. Importantly, ROC analyses indicated that arterial load accounted for most of the discriminative performance for both outcomes, whereas adding TyG to arterial load provided minimal incremental gain. Therefore, TyG appears to function primarily as a metabolic modifier rather than a dominant determinant of ambulatory BP instability in this cohort. Measures such as AASI, short-term BP variability, and morning surge reflect impaired autonomic regulation, increased vascular stiffness, and heightened sympathetic reactivity-factors strongly associated with cardiovascular morbidity and mortality [23,24,25]. Previous studies have shown that increased BP variability and blunted nocturnal dipping are linked to stroke, heart failure, and renal dysfunction, independent of mean BP levels [26,27]. Our findings reinforce the concept that ambulatory hemodynamic instability captures pathophysiological dimensions not adequately explained by traditional risk markers. Importantly, ABPM provides a dynamic assessment of blood pressure behavior that extends beyond static office measurements. Circadian patterns, short-term variability, and morning surge reflect complex interactions between vascular structure, autonomic regulation, and environmental stressors. As such, ABPM-derived phenotypes may uncover high-risk hemodynamic profiles that remain clinically silent when evaluated solely by conventional clinic blood pressure values.

A key contribution of this study is the identification of a synergistic interaction between metabolic burden and arterial instability. The combined “double-high” phenotype was associated with dramatically increased odds of both non-dipping and high systolic BP variability—far exceeding the effects of either component alone. From a pathophysiological perspective, insulin resistance is known to promote a proinflammatory and pro-oxidative vascular milieu, leading to endothelial dysfunction, impaired nitric oxide bioavailability, and increased arterial stiffness. These alterations may not uniformly translate into overt ambulatory blood pressure abnormalities; however, when combined with functional arterial instability, they may facilitate exaggerated blood pressure variability and circadian dysregulation. In this context, arterial load may be viewed as the functional conduit through which metabolic vulnerability becomes clinically manifest during daily blood pressure fluctuations. Although bias-reduced Firth penalized regression was applied to mitigate sparse-data bias, the magnitude of the observed odds ratios should be interpreted cautiously, as effect-size inflation remains possible in very small subgroups and confidence intervals remain wide. This pattern suggests a threshold phenomenon: once metabolic stress compromises endothelial and microvascular function, arterial stiffness and autonomic dysregulation may precipitate overt ambulatory BP abnormalities [28,29]. Such synergy is consistent with mechanistic studies showing that insulin resistance amplifies sympathetic tone, reduces nitric oxide bioavailability, and accelerates elastin–collagen imbalance in the arterial wall [30,31]. Under these conditions, the vasculature becomes highly sensitive to hemodynamic triggers, resulting in unstable circadian and short-term BP patterns.

The findings also provide insight into the heterogeneous cardiovascular risk trajectories observed among individuals with elevated TyG. Not all patients with insulin resistance develop severe ambulatory abnormalities, and this variability may reflect individual differences in arterial compliance and autonomic responsiveness [32]. Our results suggest that arterial load may represent a complementary or amplifying factor that is associated with the manifestation of clinically meaningful ambulatory BP abnormalities in metabolically vulnerable individuals.

From a clinical perspective, the double-high phenotype may represent a previously unrecognized high-risk endotype within the hypertensive population. Patients with this phenotype demonstrated profound nocturnal BP loss, extreme BP variability, and markedly exaggerated morning surge—patterns known to predict stroke, acute coronary events, and cognitive decline [33,34]. These findings suggest potential applications for targeted ABPM-based risk stratification and consideration of individualized chronotherapy or intensive lifestyle/metabolic interventions.

Nonetheless, several considerations must temper these interpretations. The cross-sectional design precludes causal inference, and longitudinal studies are required to determine whether the double-high phenotype predicts incident cardiovascular events or therapeutic responsiveness. Additionally, arterial load was defined using ABPM-derived functional measures rather than imaging-based stiffness indices. However, these metrics have been shown to correlate strongly with vascular dysfunction and are widely used in clinical and research settings.

In summary, the coexistence of high metabolic burden and high arterial instability identifies a distinct and particularly high-risk ambulatory blood pressure phenotype. Accordingly, any potential clinical applications should be interpreted as exploratory and hypothesis-generating, rather than as direct therapeutic recommendations. Metabolic burden alone is insufficient to generate adverse ABPM features unless accompanied by impaired vascular and autonomic regulation. Integrating TyG with ABPM-derived arterial load measures enables refined risk stratification and reveals a synergistic pathway linking insulin resistance biology to circadian and dynamic BP dysregulation.

## 5. Clinical Implications

The findings of this study highlight a clinically meaningful endotype in which metabolic burden and arterial instability converge to produce severe ambulatory blood pressure disruption. Routine use of the TyG index together with ABPM-derived arterial load metrics may enable earlier identification of individuals at disproportionately high hemodynamic risk despite modest clinical blood pressure levels. Patients classified as having the double-high phenotype—though relatively uncommon—demonstrate profound nocturnal BP dysregulation and extreme short-term variability, patterns strongly linked to stroke, acute coronary events, and target-organ damage.

Recognition of this phenotype may help identify individuals who warrant closer ambulatory monitoring and further metabolic evaluation. Potential implications for personalized treatment strategies remain hypothesis-generating and should be explored in longitudinal and interventional studies. Integrating metabolic and hemodynamic assessments may therefore refine risk stratification beyond conventional clinic BP measurements and support a more precise, mechanism-oriented approach to hypertension management. In clinical practice, such integrative phenotyping may be particularly relevant for patients with apparently controlled office blood pressure but persistent cardiometabolic risk. Identifying individuals with concurrent metabolic vulnerability and arterial instability may prompt closer ambulatory surveillance and reinforce the importance of comprehensive risk assessment that incorporates both metabolic and hemodynamic domains. Nevertheless, these potential applications should be interpreted cautiously and primarily serve as a framework for future prospective and interventional research.

## 6. Limitations

This study has several limitations that should be acknowledged. First, its cross-sectional and retrospective design precludes any causal inference regarding the temporal relationship between metabolic burden, arterial load, and ambulatory blood pressure abnormalities. Longitudinal cohort studies are required to determine whether the double-high phenotype predicts incident cardiovascular events or progression of hypertension.

Second, arterial load was defined using ABPM-derived functional indices (AASI, BP variability, and morning surge) rather than imaging-based or invasive measures of vascular structure. Although these parameters are validated surrogates of vascular dysfunction, they may not fully capture structural arterial changes.

Third, the number of individuals classified as having the double-high phenotype was relatively small. This reflects the true rarity of this extreme-risk subgroup but may have contributed to quasi-complete separation in logistic regression models and to wide confidence intervals, despite the use of bias-reduced Firth estimation.

Fourth, insulin resistance was estimated using the TyG index rather than gold-standard methods such as the hyperinsulinemic-euglycemic clamp. Although TyG is widely accepted and highly correlated with insulin resistance, some degree of misclassification is possible and would likely bias results toward the null.

Fifth, antihypertensive medications were included as a binary variable, without detailed stratification by drug class, dose, or chronotherapy regimen. Residual confounding related to pharmacologic effects on circadian blood pressure patterns cannot be excluded.

Finally, this was a single-center study conducted in a specific regional population, which may limit generalizability. In addition, because patients with established cardiovascular disease were excluded, the findings may not directly generalize to higher-risk populations routinely encountered in tertiary cardiovascular clinics. External validation in independent cohorts with more diverse demographic and clinical characteristics is warranted.

## 7. Conclusions

In this ABPM-based cohort, the coexistence of elevated metabolic burden and increased arterial load identified a distinct hemodynamic endotype characterized by markedly impaired nocturnal blood pressure decline and exaggerated short-term systolic variability. While the TyG index alone demonstrated limited discriminatory capacity for adverse ambulatory phenotypes, arterial load emerged as the principal driver of circadian and dynamic BP instability. The combined “double-high” phenotype exhibited a dramatically amplified risk profile, suggesting a synergistic interaction between metabolic vulnerability and functional arterial dysfunction.

These findings indicate that metabolic stress translates into clinically significant ambulatory blood pressure derangement primarily in the presence of an unstable arterial substrate. Integrating TyG with ABPM-derived arterial load metrics may therefore improve risk stratification and help identify a small but clinically meaningful subgroup of patients who may benefit from intensified follow-up, more comprehensive metabolic evaluation, and individualized therapeutic strategies.

## Figures and Tables

**Table 1:** Baseline clinical, metabolic, and ambulatory blood pressure characteristics of the study population.

Variable	Total Population (*n* = 294)
Age, years	51.8 ± 11.7
Male sex, *n* (%)	146 (49.7%)
Diabetes mellitus, *n* (%)	26 (8.8%)
Antihypertensive treatment, *n* (%)	114 (38.8%)
Fasting plasma glucose, mg/dL	98.3 ± 15.5
Triglycerides, mg/dL	155.8 ± 59.9
HDL-C, mg/dL	47.7 ± 9.9
LDL-C, mg/dL	121.5 ± 28.5
Total cholesterol, mg/dL	200.4 ± 32.4
TyG index	8.86 ± 0.41
24 h mean SBP, mmHg	131.4 ± 11.7
24 h mean DBP, mmHg	79.2 ± 8.3
Daytime SBP, mmHg	133.4 ± 12.2
Nighttime SBP, mmHg	115.6 ± 11.8
Daytime DBP, mmHg	80.1 ± 8.8
Nighttime DBP, mmHg	69.7 ± 8.6
Nocturnal dipping (% drop)	12.0 ± 5.4
AASI	0.45 ± 0.09
24 h SBP ARV	12.23 ± 3.16
24 h DBP ARV	8.76 ± 2.38
Smoothness index	1.10 ± 0.11
Morning surge, mmHg	17.5 ± 5.6
Non-dipper pattern, *n* (%)	87 (29.6%)
High SBP variability, *n* (%)	87 (29.6%)
High AASI, *n* (%)	45 (15.3%)
Low smoothness index, *n* (%)	9 (3.1%)
High morning surge, *n* (%)	36 (12.2%)
TyG quartiles (Q1–Q4), *n* (%)	Q1: 75 (25.5%)/Q2: 72 (24.5%) /Q3: 73 (24.8%)/Q4: 74 (25.2%)

**Table 2:** Comparison of metabolic and ambulatory blood pressure parameters between double-high and non-double-high groups.

Parameter	Others (Non-Double-High) (*n* = 279)	Double-High (*n* = 15)	*p* Value
Age, years	51.5 ± 11.8	54.7 ± 10.3	0.168
Fasting plasma glucose, mg/dL	97.2 ± 13.8	117.4 ± 24.3	<0.001
Triglycerides, mg/dL	151.0 ± 57.6	227.4 ± 64.6	<0.001
HDL-C, mg/dL	47.8 ± 9.8	48.8 ± 11.1	0.704
LDL-C, mg/dL	121.1 ± 28.3	122.3 ± 29.7	0.874
TyG index	8.82 ± 0.40	9.42 ± 0.28	<0.001
24 h mean SBP, mmHg	131.0 ± 11.7	137.1 ± 10.9	0.051
24 h mean DBP, mmHg	79.1 ± 8.3	80.5 ± 8.1	0.530
Daytime SBP, mmHg	132.9 ± 12.3	139.0 ± 11.1	0.060
Nighttime SBP, mmHg	114.2 ± 10.9	126.0 ± 13.1	<0.001
Daytime DBP, mmHg	80.1 ± 8.9	80.6 ± 8.4	0.842
Nighttime DBP, mmHg	69.1 ± 8.3	74.6 ± 9.4	0.016
Nocturnal dipping, %	12.7 ± 5.0	8.0 ± 5.0	0.001
AASI	0.44 ± 0.08	0.55 ± 0.06	<0.001
24 h SBP-ARV, mmHg	11.80 ± 2.98	15.69 ± 1.89	<0.001
24 h DBP-ARV, mmHg	8.57 ± 2.33	10.16 ± 2.26	0.011
Smoothness index	1.10 ± 0.11	1.08 ± 0.11	0.474
Morning surge, mmHg	16.95 ± 5.29	23.62 ± 5.41	<0.001
High morning surge, *n* (%)	27/279 (9.7%)	9/15 (60.0%)	<0.001
Non-dipper pattern, *n* (%)	76/279 (27.2%)	11/15 (73.3%)	<0.001
High SBP-ARV, *n* (%)	73/279 (26.2%)	14/15 (93.3%)	<0.001

**Table 3:** Multivariable logistic regression analysis for predictors of non-dipping blood pressure pattern.

Variable	Adjusted OR	95% CI	*p* Value
Double-high phenotype	42.0	5.18–341.0	<0.001
Age	1.00	0.98–1.02	0.995
Male sex	1.66	0.98–2.83	0.062
Diabetes	0.33	0.10–1.13	0.078
Antihypertensive treatment	0.65	0.37–1.15	0.136

**Table 4:** Penalized (Firth) multivariable logistic regression analysis for the non-dipping blood pressure pattern.

Variable	OR	95% CI	*p*-Value
Double-high phenotype	11.73	3.61–47.63	<0.001
Age (per year)	0.99	0.97–1.01	0.498
Male sex	1.64	0.97–2.79	0.063
Diabetes mellitus	0.37	0.10–1.09	0.073
Antihypertensive treatment	0.66	0.37–1.14	0.139

Likelihood ratio test = 22.89, *p* = 0.00035, Wald test = 58.09, *p* < 0.001, *n* = 294.

**Table 5:** Multivariable logistic regression analysis for predictors of high systolic blood pressure variability.

Variable	Adjusted OR	95% CI	*p* Value
Double-high phenotype	41.7	6.02–289.3	<0.001
Age	1.00	—	>0.05
Male sex	1.11	—	>0.05
Diabetes	0.75	—	>0.05
Antihypertensive treatment	1.21	—	>0.05

**Table 6:** Penalized (Firth) multivariable logistic regression analysis for high 24 h systolic blood pressure variability.

Variable	OR	95% CI	*p* -Value
Double-high phenotype	26.29	6.15–246.70	<0.001
Age (per year)	1.00	0.98–1.02	0.989
Male sex	1.11	0.65–1.89	0.706
Diabetes mellitus	0.80	0.26–2.08	0.654
Antihypertensive treatment	1.21	0.70–2.07	0.499

Likelihood ratio test = 28.35, *p* = 3.1 × 10^−5^, Wald test = 64.48, *p* < 0.001, *n* = 294.

**Table 7:** ROC analysis comparing predictive models for non-dipping blood pressure pattern and high systolic BP variability (SBP-ARV).

Outcome	Model	AUC	*p*
Non-dipping pattern	Model A: Clinical variables	0.585	0.021
	Model B: Clinical + TyG	0.623	0.001
	Model C: Clinical + Arterial Load	0.819	<0.001
	Model D: Clinical + TyG + Arterial Load	0.821	<0.001
High SBP Variability (ARV)	Model A: Clinical variables	0.551	0.171
	Model B: Clinical + TyG	0.577	0.038
	Model C: Clinical + Arterial Load	0.979	<0.001
	Model D: Clinical + TyG + Arterial Load	0.978	<0.001

## Data Availability

The datasets generated and/or analyzed during the current study are not publicly available due to institutional restrictions on sharing patient-level clinical data, but are available from the corresponding author (A.Y.) on reasonable request and with the permission of Karamanoğlu Mehmetbey University Faculty of Medicine.

## Abbreviations

AASI	Ambulatory Arterial Stiffness Index
ABPM	Ambulatory Blood Pressure Monitoring
ARV	Average Real Variability
AUC	Area Under the Curve
BP	Blood Pressure
DBP	Diastolic Blood Pressure
FPG	Fasting Plasma Glucose
HDL-C	High-Density Lipoprotein Cholesterol
LDL-C	Low-Density Lipoprotein Cholesterol
OR	Odds Ratio
PWV	Pulse Wave Velocity
ROC	Receiver Operating Characteristic
SBP	Systolic Blood Pressure
SD	Standard Deviation
SI	Smoothness Index
TyG	Triglyceride–Glucose Index
TyG_z	Z-standardized Triglyceride–Glucose Index
